# Supplementary figures and images for: RIdeogram: drawing SVG graphics to visualize and map genome-wide data on the idiograms
Source: PeerJ Comput Sci. 2020 Jan 20;6:e251. doi: 10.7717/peerj-cs.251 (PMC7924719; doi:10.7717/peerj-cs.251)

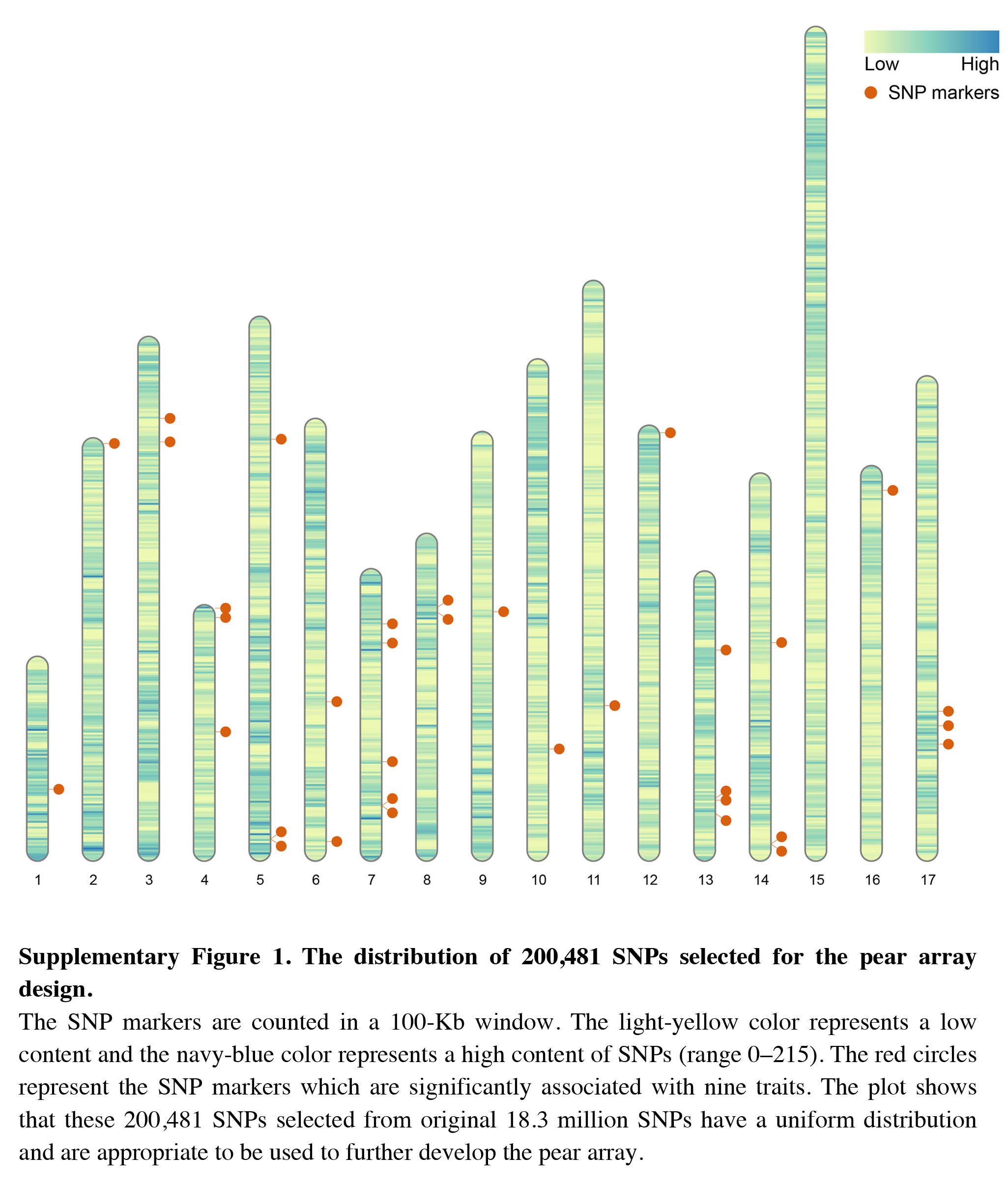

Supplement: Figure S1 — The SNP markers are counted in a 100-Kb window. The light-yellow color represents a low content and the navy-blue color represents a high content of SNPs (range 0–215). The red circles represent the SNP markers which are significantly associated with nine traits. The plot shows that these 200,481 SNPs selected from original 18.3 million SNPs have a uniform distribution and are appropriate to be used to further develop the pear array. [file peerj-cs-06-251-s001.png]

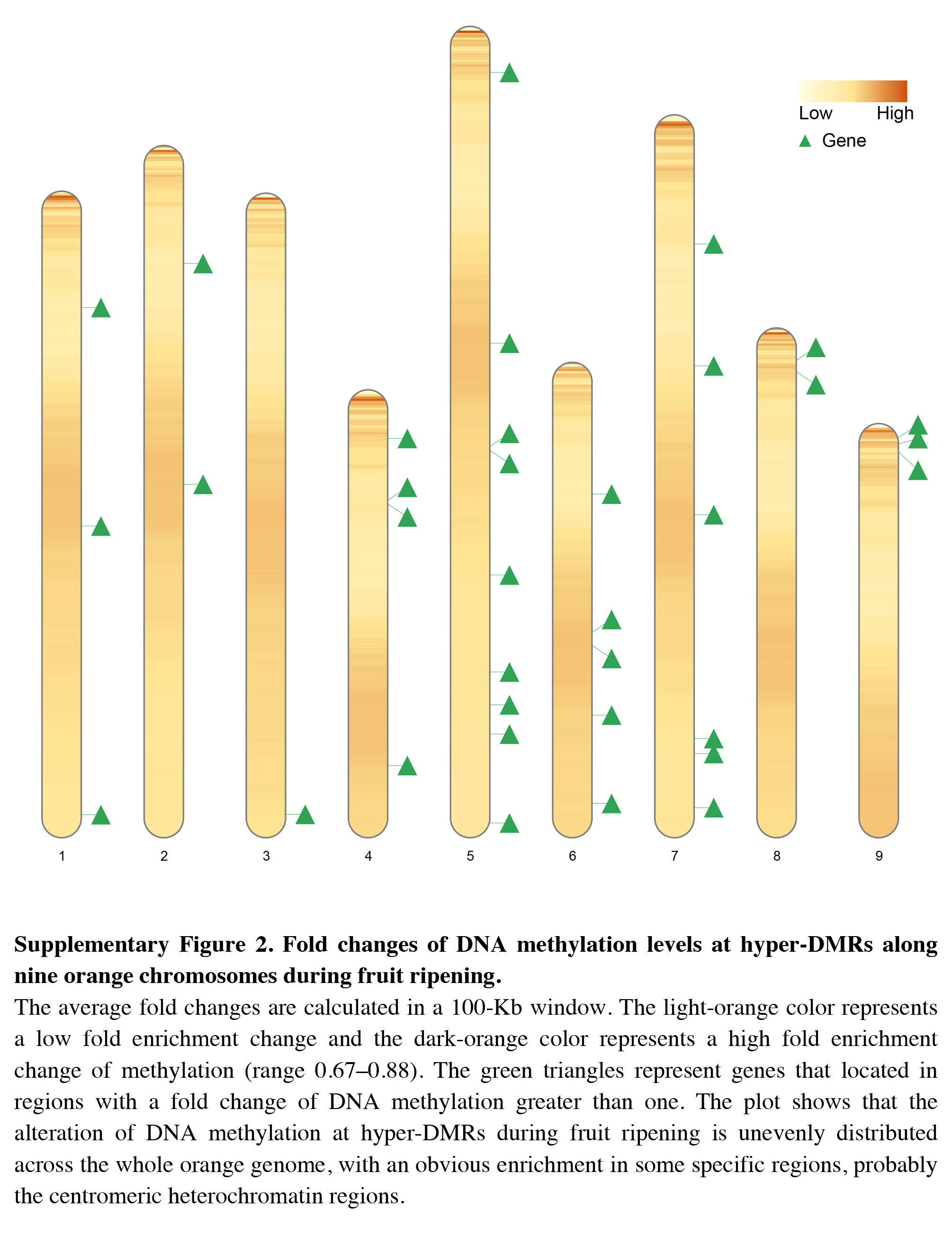

Supplement: Figure S2 — The average fold changes are calculated in a 100-Kb window. The light-orange color represents a low fold enrichment change and the dark-orange color represents a high fold enrichment change of methylation (range 0.67–0.88). The green triangles represent genes that located in regions with a fold change of DNA methylation greater than one. The plot shows that the alteration of DNA methylation at hyper-DMRs during fruit ripening is unevenly distributed across the whole orange genome, with an obvious enrichment in some specific regions, probably the centromeric heterochromatin regions. [file peerj-cs-06-251-s002.png]

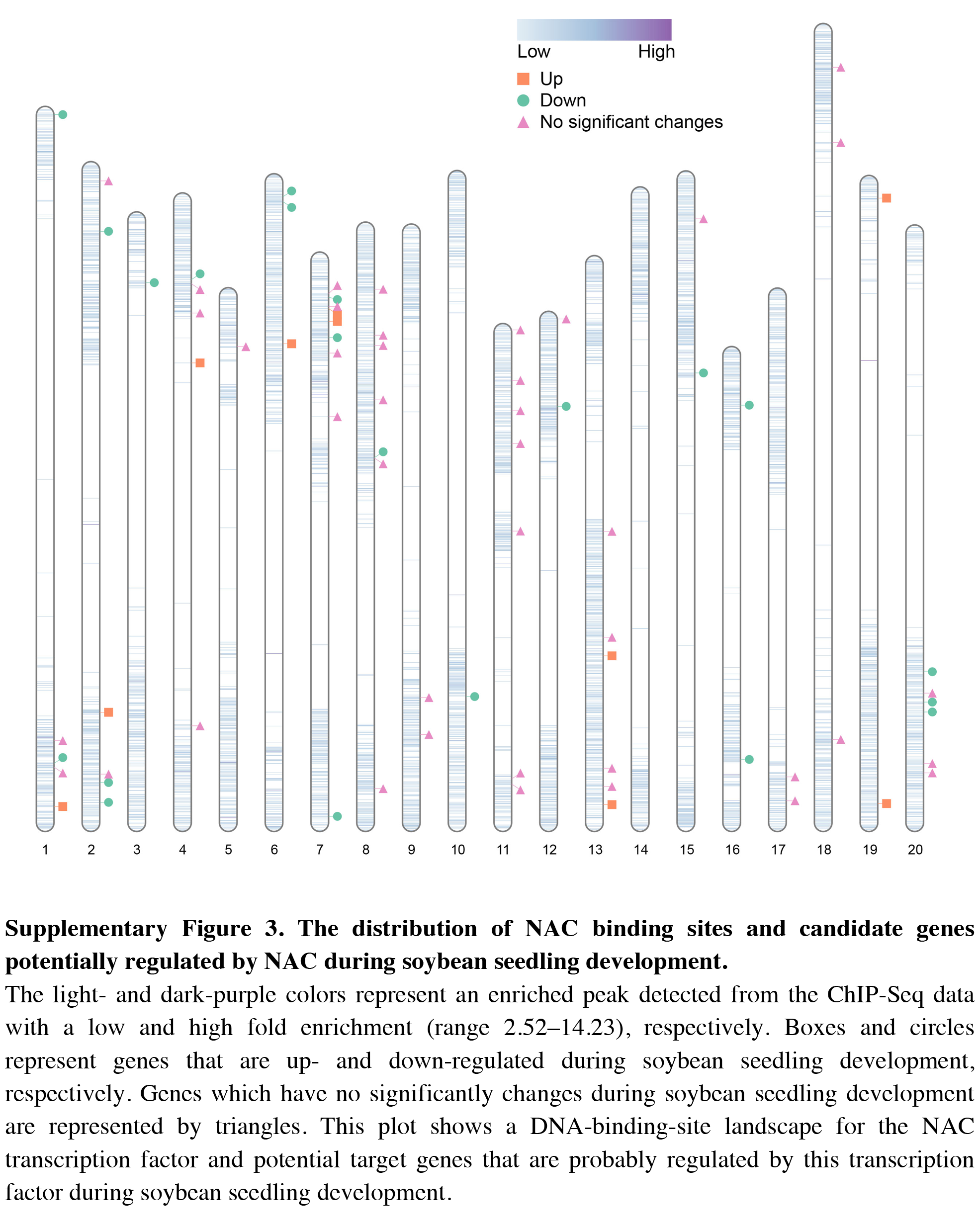

Supplement: Figure S3 — The light- and dark-purple colors represent an enriched peak detected from the ChIP-Seq data with a low and high fold enrichment (range 2.52–14.23), respectively. Boxes and circles represent genes that are up- and down-regulated during soybean seedling development, respectively. Genes which have no significantly changes during soybean seedling development are represented by triangles. This plot shows a DNA-binding-site landscape for the NAC transcription factor and potential target genes that are probably regulated by this transcription factor during soybean seedling development. [file peerj-cs-06-251-s003.png]

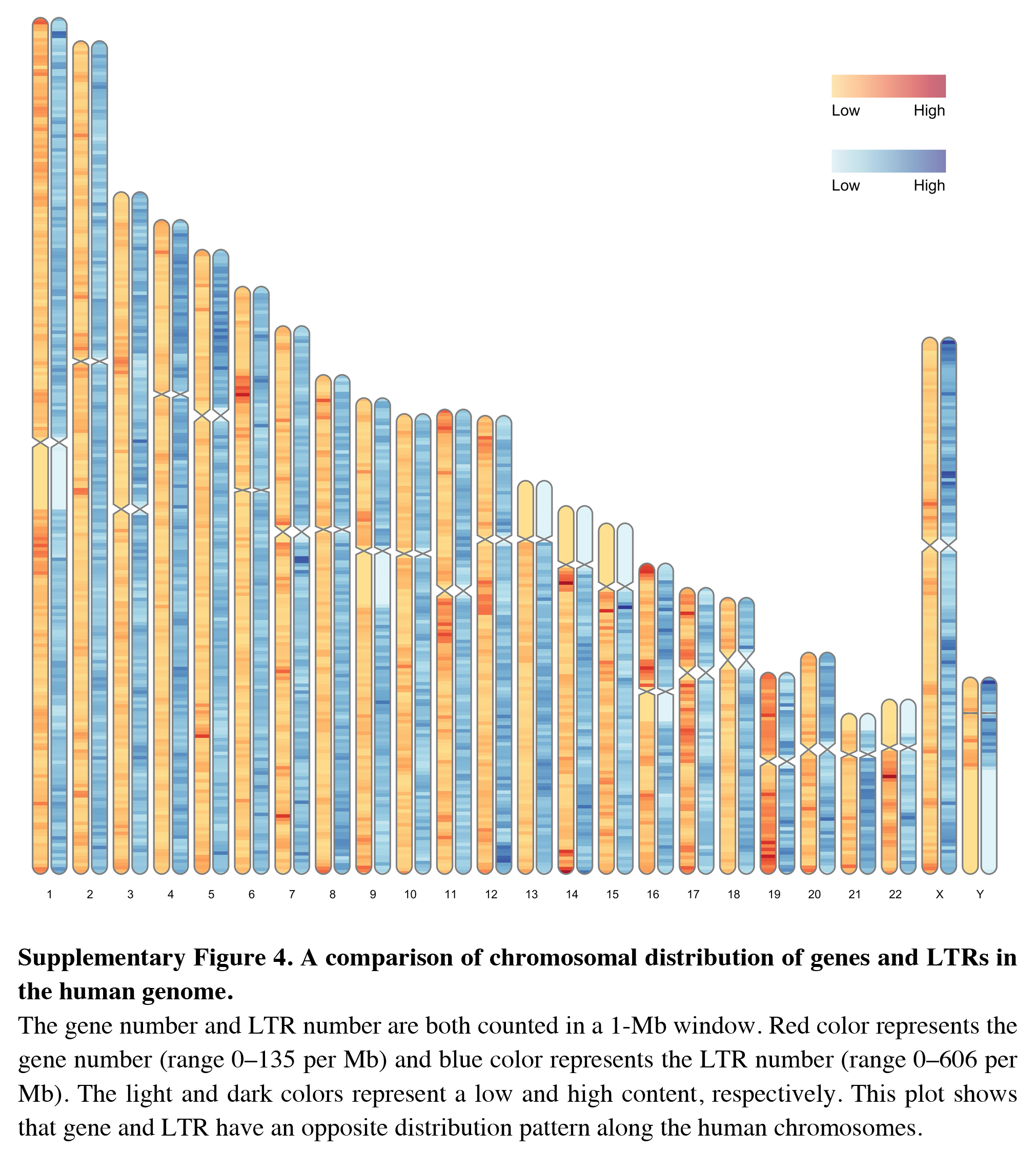

Supplement: Figure S4 — The gene number and LTR number are both counted in a 1-Mb window. Red color represents the gene number (range 0–135 per Mb) and blue color represents the LTR number (range 0–606 per Mb). The light and dark colors represent a low and high content, respectively. This plot shows that gene and LTR have an opposite distribution pattern along the human chromosomes. [file peerj-cs-06-251-s004.png]

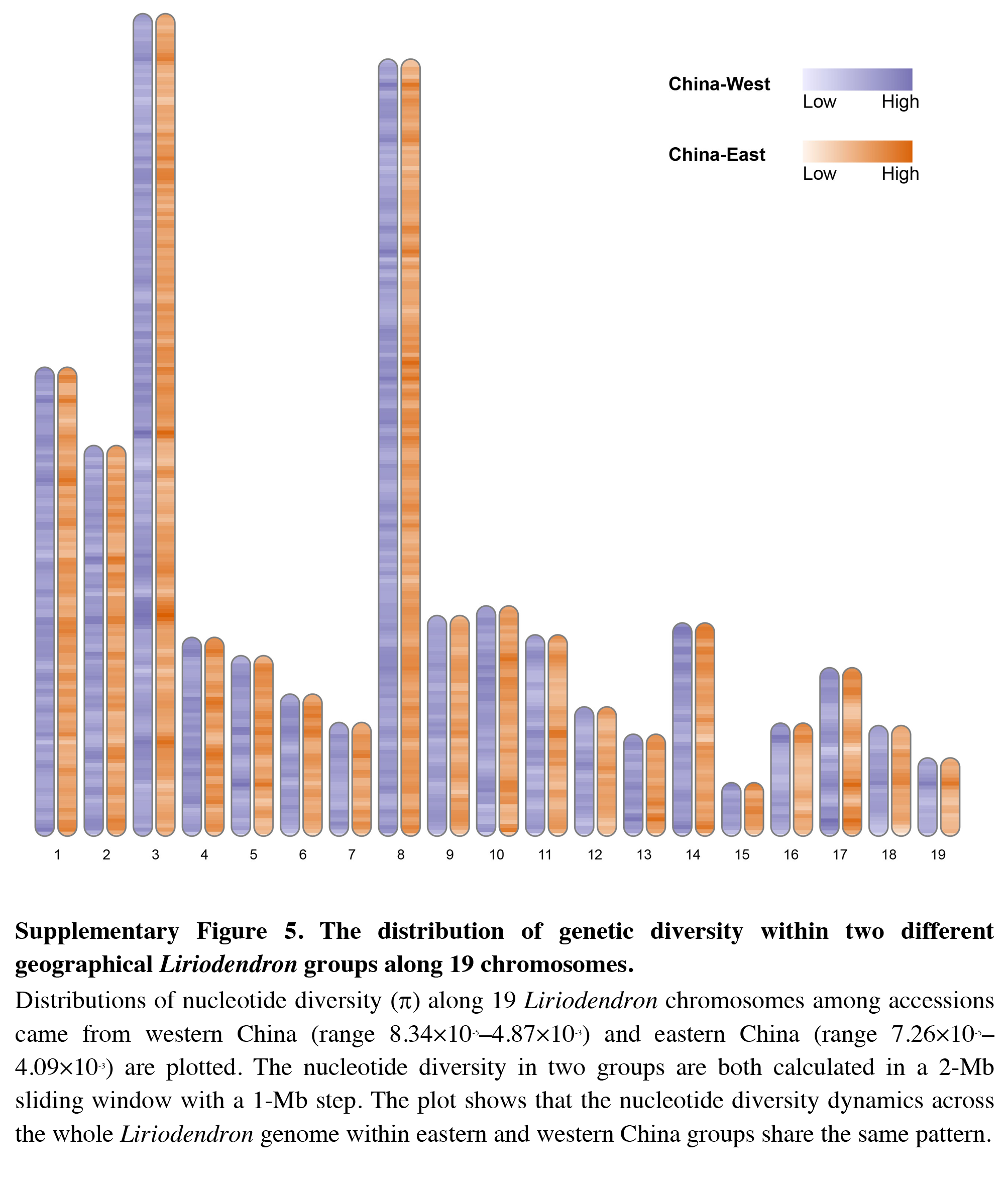

Supplement: Figure S5 — Distributions of nucleotide diversity (p) along 19 Liriodendron chromosomes among accessions came from western (range 8.34 × 10^−5^–4.87 × 10^−3^) and eastern China (range 7.26 × 10^−5^–4.09 × 10^−3^) are plotted. The nucleotide diversity in two groups are both calculated in a 2-Mb sliding window with a 1-Mb step. The plot shows that the nucleotide diversity dynamics across the whole Liriodendron genome within eastern and western China groups share the same pattern. [file peerj-cs-06-251-s005.png]

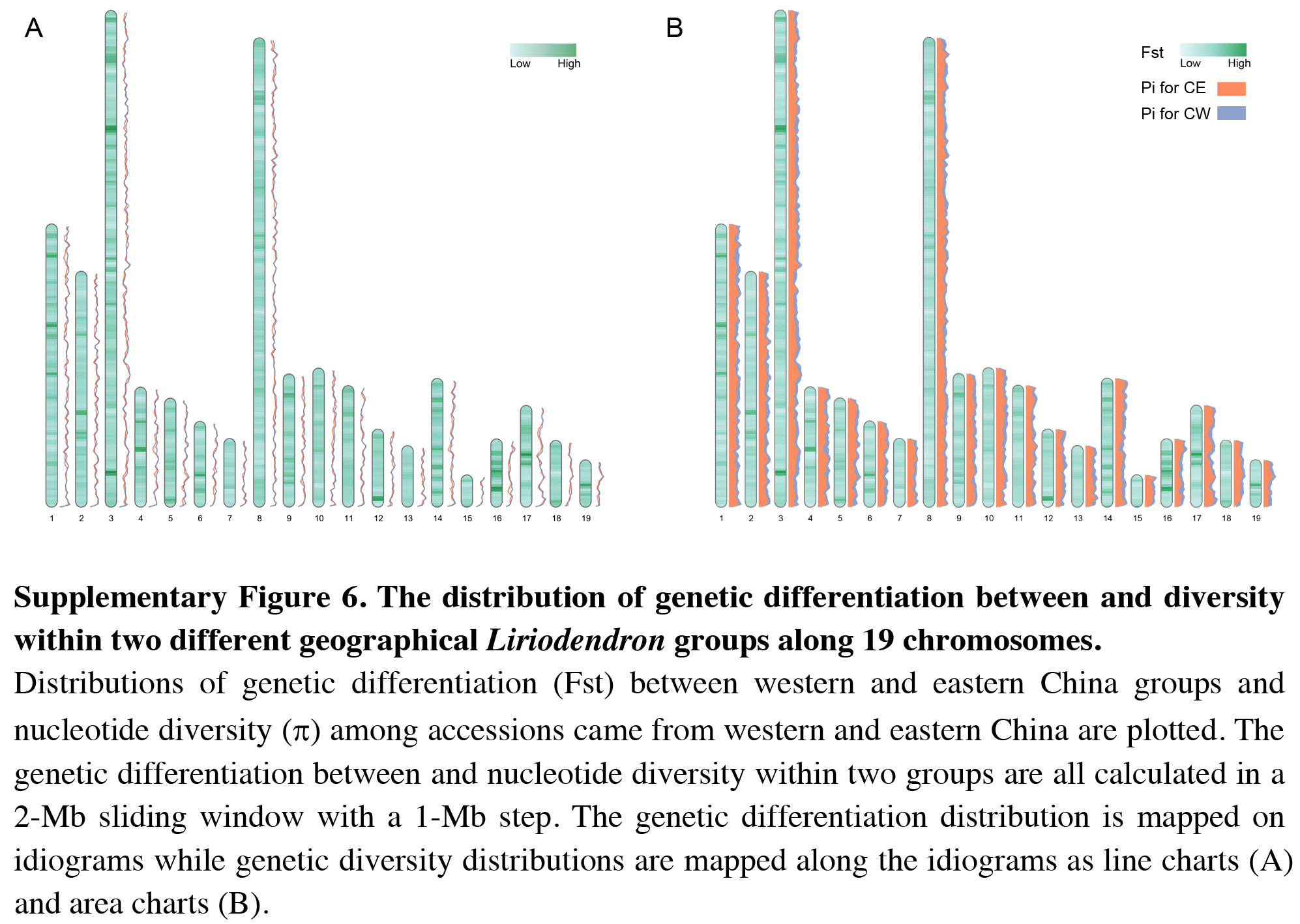

Supplement: Figure S6 — Distributions of genetic differentiation (Fst) between western and eastern China groups and nucleotide diversity (p) among accessions came from western and eastern China are plotted. The genetic differentiation between and nucleotide diversity within two groups are all calculated in a 2-Mb sliding window with a 1-Mb step. The genetic differentiation distribution is mapped on idiograms while genetic diversity distributions are mapped along the idiograms as line charts (a) and area charts (b). [file peerj-cs-06-251-s006.png]

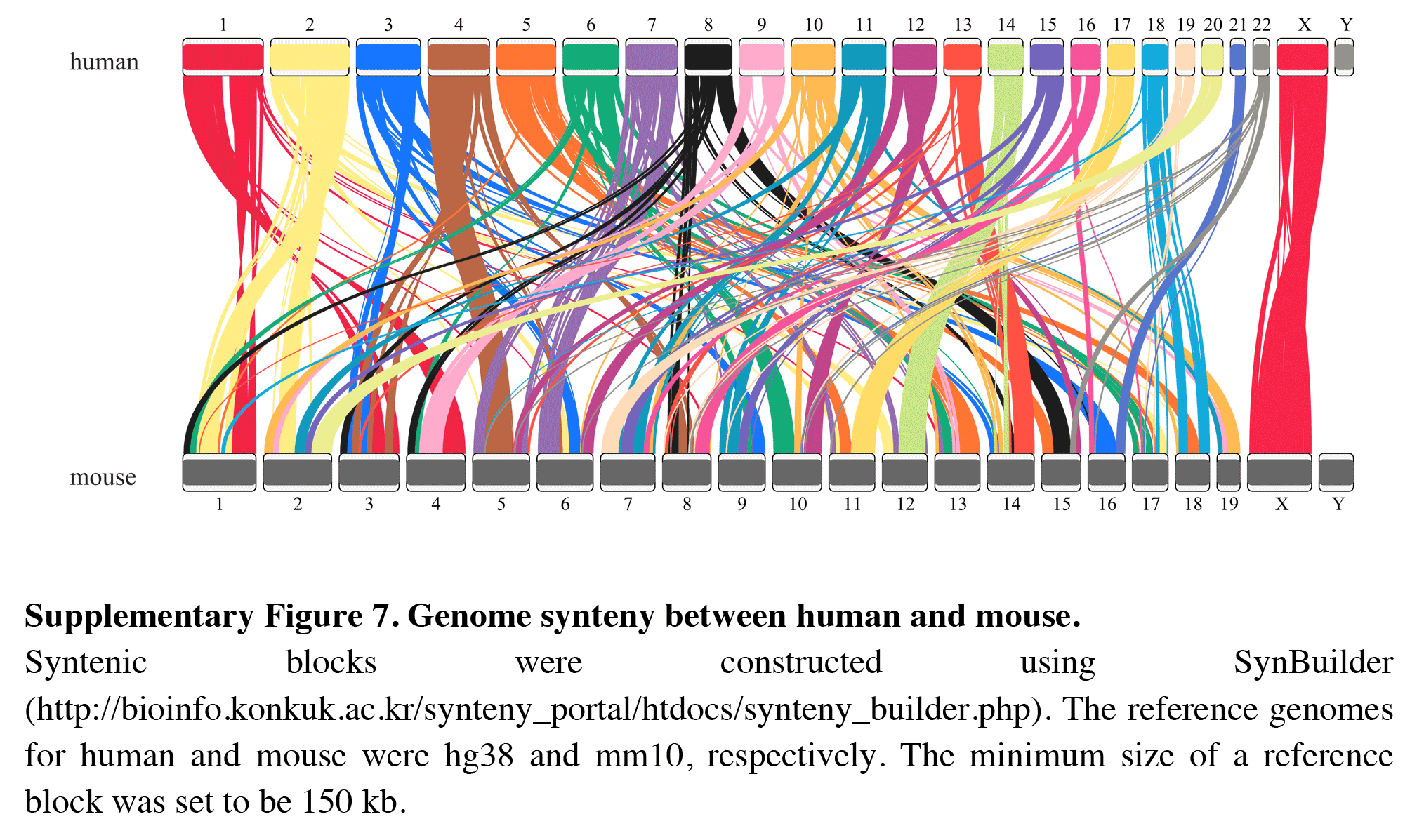

Supplement: Figure S7 — Syntenic blocks were constructed using SynBuilder ( http://bioinfo.konkuk.ac.kr/synteny_portal/htdocs/synteny_builder.php). The reference genomes for human and mouse were hg38 and mm10, respectively. The minimum size of a reference block was set to be 150 kb. [file peerj-cs-06-251-s007.png]

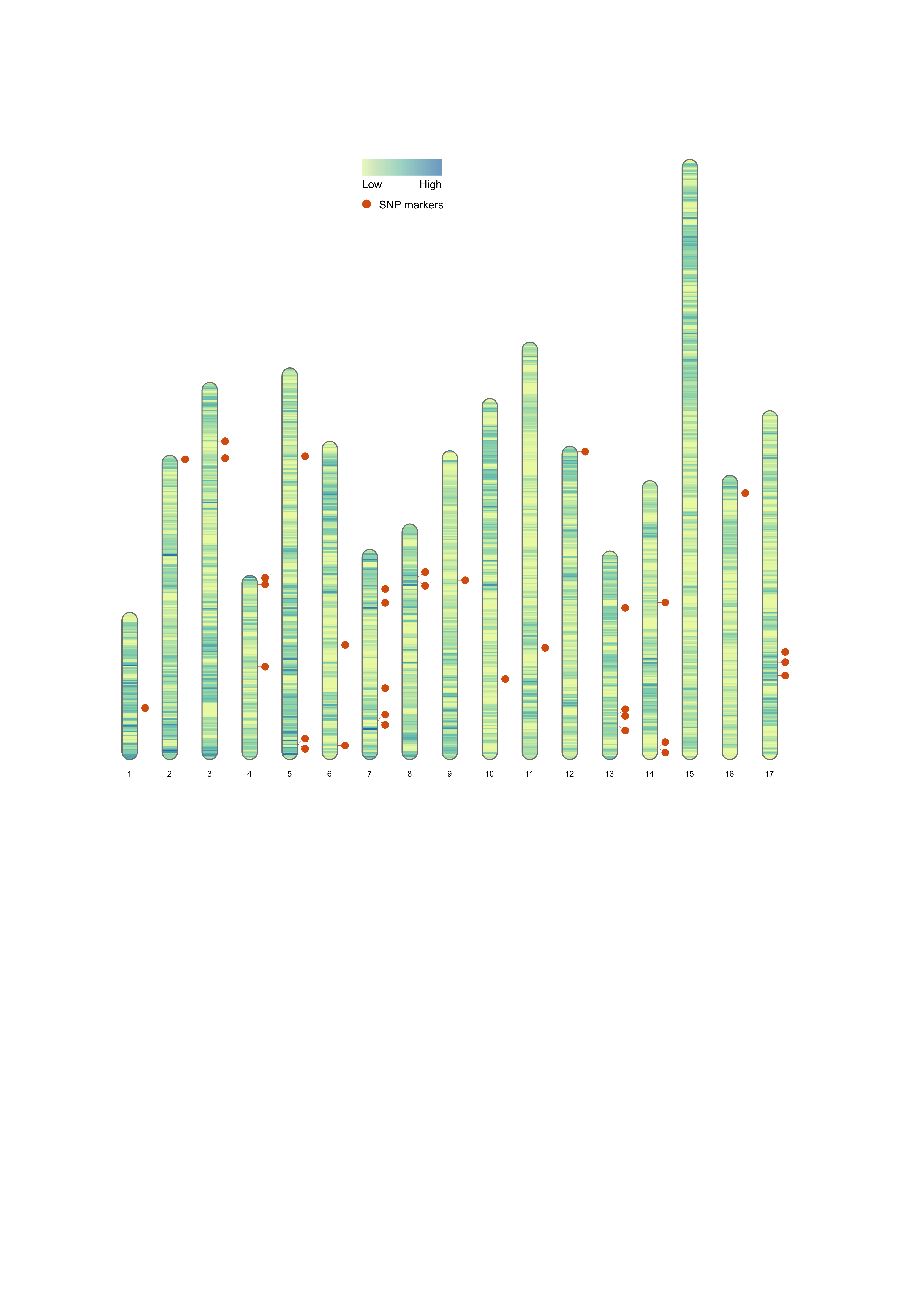

Supplement: Data S1 [file peerj-cs-06-251-s008.zip › Supplemental Data S1/chromosome.png]

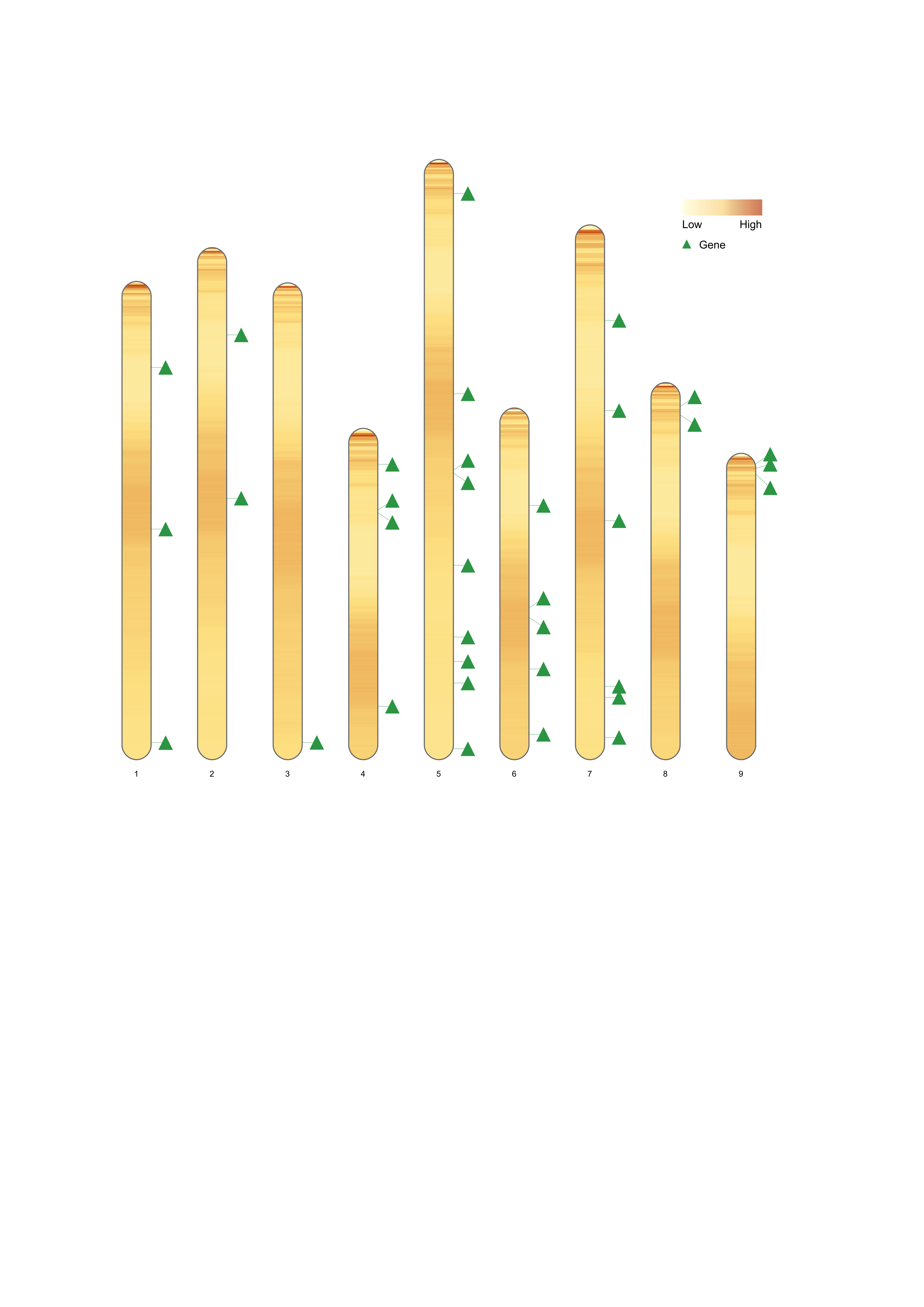

Supplement: Data S2 [file peerj-cs-06-251-s009.zip › Supplemental Data S2/chromosome.png]

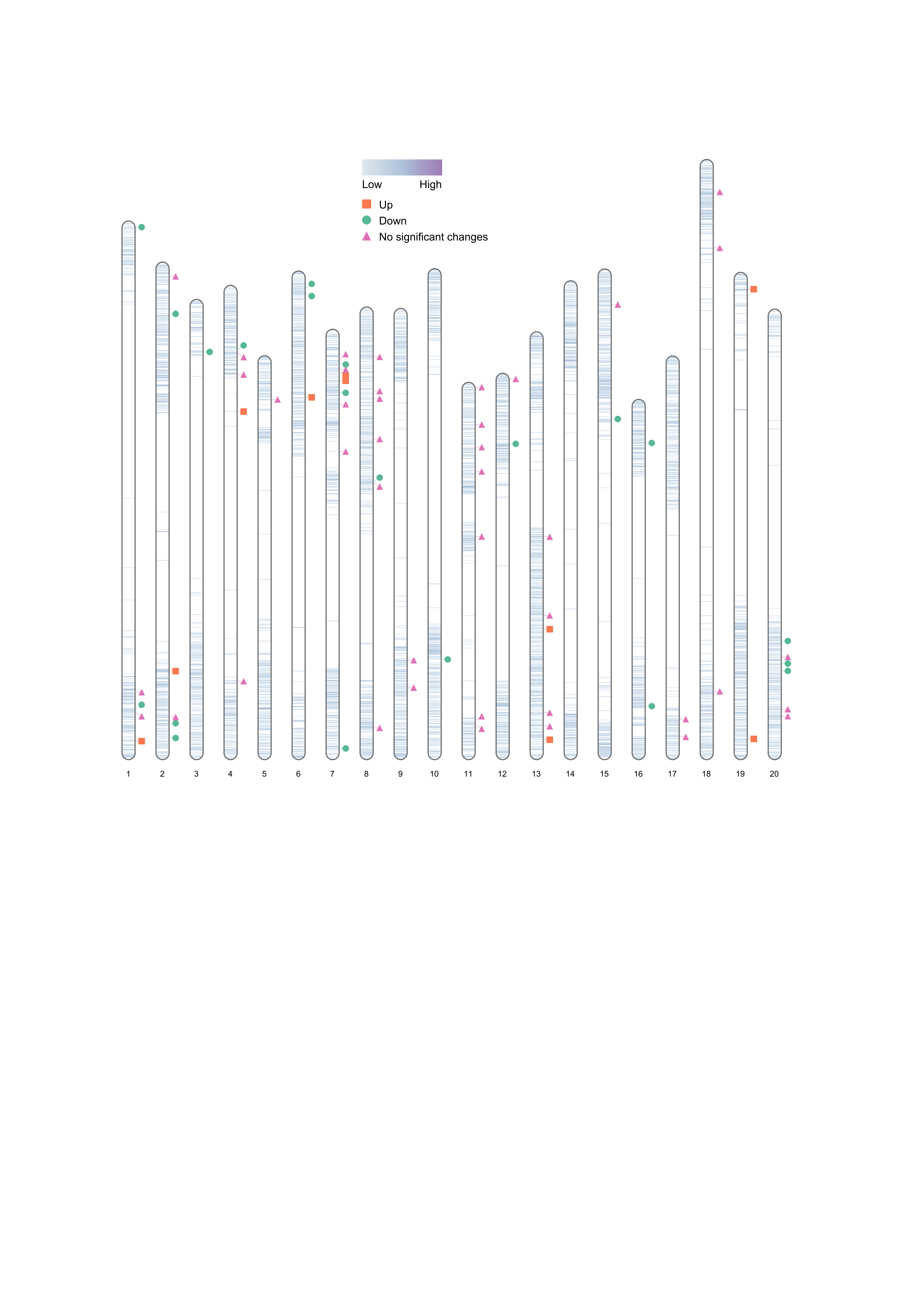

Supplement: Data S3 [file peerj-cs-06-251-s010.zip › Supplemental Data S3/chromosome.png]

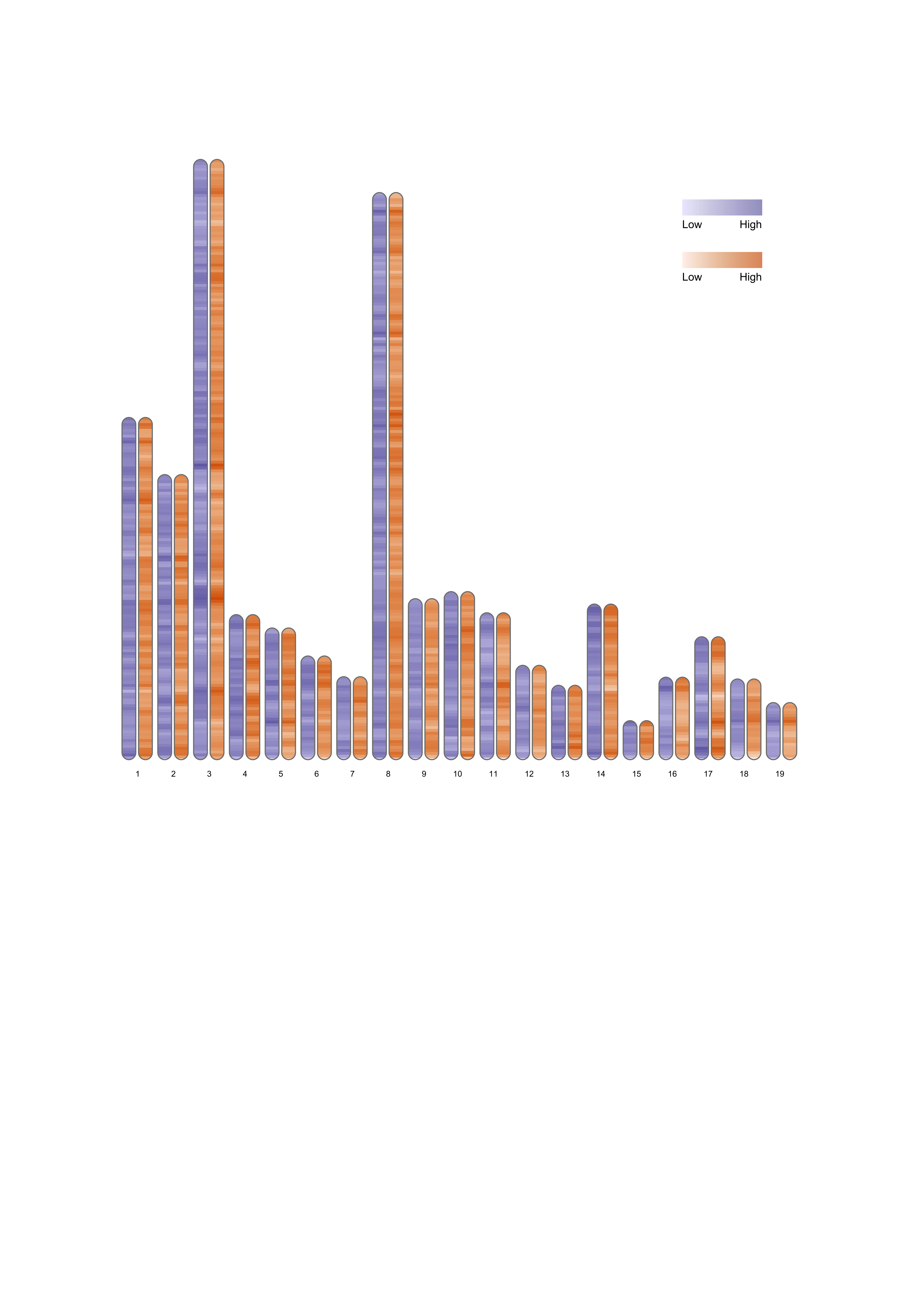

Supplement: Data S4 [file peerj-cs-06-251-s011.zip › Supplemental Data S4/chromosome.png]

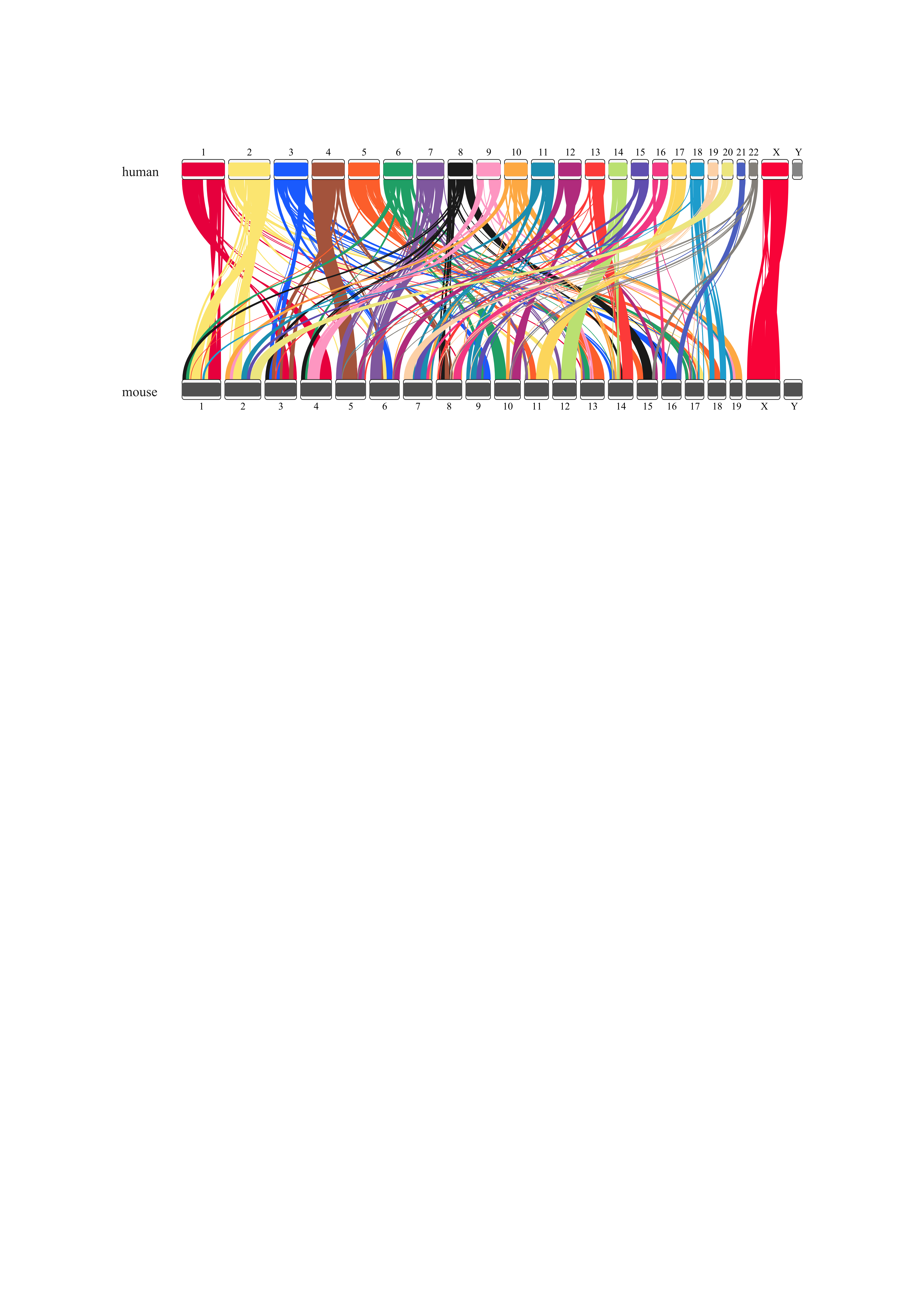

Supplement: Data S5 [file peerj-cs-06-251-s012.zip › Supplemental Data S5/chromosome.png]
