# Supplementary material for: RIdeogram: drawing SVG graphics to visualize and map genome-wide data on the idiograms
Source: PeerJ Comput Sci. 2020 Jan 20;6:e251. doi: 10.7717/peerj-cs.251 (PMC7924719; doi:10.7717/peerj-cs.251)
Supplement: Article S1 [file peerj-cs-06-251-s013.pdf]

Codes for examples

The codes used to get the Figs. 1, 2, S4 and S6 using example data contained in the package *RIdeogram*. For more detailed information about the parameter settings and general applications of this package, users can refer to the tutorial vignette (vignette("RIdeogram")).

Figure 1

First, we load the package:

```
> library(RIdeogram)
```

Then, we load the human karyotype information, gene density data and positions of 500 random RNAs:

```
> data(human_ karyotype, package = "RIdeogram")
> data(gene_density, package = "RIdeogram")
> data(Random_RNAs_500, package = "RIdeogram")
```

Next, we can use the function to *ideogram* get the SVG file:

```
> ideogram(karyotype = human_karyotype, overlaid = gene_density, label =
Random_RNAs_500, label_type = “marker”)
```

And, we will get a SVG file in the current working directory. We can get the current working directory information using the following code:

```
> getwd()
```

We can use the software Inkscape to view this SVG file that contains a vector graphic. Or, we can use the function *convertSVG* to convert this SVG file into an image file:

```
> convertSVG("chromosome.svg", device = "png")
```

Finally, we will get the gene distribution plot which is displayed in Fig. 1.

Figure 2

First, we load the package:

```
> library(RIdeogram)
```

Then, we load the karyotype information for three plants, ternary genome synteny data:

```
> data(karyotype_ternary_comparison, package="RIdeogram")
> data(synteny_ternary_comparison_graident, package = "RIdeogram")
```

Next, we use the function to *ideogram* get the SVG file:

```
> ideogram(karyotype = karyotype_ternary_comparison, synteny =
synteny_ternary_comparison_graident)
```

After that, we use the function *rsvg_pdf* from the package *rsvg* to convert this SVG file into a pdf file:

```
> library("rsvg")
> rsvg_pdf("chromosome.svg", "chromosome.pdf")
```

Finally, we get the genome synteny plot of three plant genomes which is displayed in Fig. 2.

Figure S4
First, we load the package:

```
> library(RIdeogram)
```

Then, we load the human karyotype information, gene and LTR density data:

```
> data(human_ karyotype, package = "RIdeogram")
> data(gene_density, package = "RIdeogram")
> data(LTR_density, package = "RIdeogram")
```

Next, we use the function to *ideogram* get the SVG file:

```
> ideogram(karyotype = human_karyotype, overlaid = gene_density, label = LTR_density,
label_type = “heatmap”, colorset1 = c("#fee090", "#fdae61", "#f46d43", "#d73027", "#a50026"),
colorset2 = c("#e0f3f8", "#abd9e9", "#74add1", "#4575b4", "#313695"))
```

After that, we use the function *convertSVG* to convert this SVG file into an image file:

```
> convertSVG("chromosome.svg", device = "png")
```

Finally, we get the comparison plot of gene and LTR distribution which is displayed in Fig. S4.

Figure S6
First, we load the package:

```
> library(RIdeogram)
```

Then, we load the *Liriodendron* karyotype information, genetic differentiation and diversity data:

```
> data(liriodendron_karyotype, package="RIdeogram")
> data(Fst_between_CE_and_CW, package="RIdeogram
> data(Pi_for_CE_and_CW, package="RIdeogram")
```

Next, we use the function to *ideogram* get the SVG file:

```
> ideogram(karyotype = liriodendron_karyotype, overlaid = Fst_between_CE_and_CW, label =
Pi_for_CE_and_CW, label_type = "line", colorset1 = c("#e5f5f9", "#99d8c9", "#2ca25f"))
```

After that, we use the function *convertSVG* to convert this SVG file into an image file:

```
> convertSVG("chromosome.svg", device = "png")
```

Finally, we get Fig. S6A.

And for Fig. S6B, we use the same data as above.

So, we can use the function to *ideogram* get the SVG file:

```
> ideogram(karyotype = liriodendron_karyotype, overlaid = Fst_between_CE_and_CW, label =
Pi_for_CE_and_CW, label_type = "polygon", colorset1 = c("#e5f5f9", "#99d8c9", "#2ca25f"))
```

After that, we use the function *convertSVG* to convert this SVG file into an image file:

```
> convertSVG("chromosome.svg", device = "png")
```

Finally, we get Fig. S6B.
